# RESEARCH ISSUES AND INITIATIVES: Cancer Report Examines Environmental Hazards

**DOI:** 10.1289/ehp.118-a336a

**Published:** 2010-08

**Authors:** Catherine M. Cooney

**Affiliations:** **Catherine M. Cooney**, a science writer in Washington, DC, has written for *Environmental Science & Technology* and *Chemical Watch*

In its new report, *Reducing Environmental Cancer Risk: What We Can Do Now*, the President’s Cancer Panel (PCP) for the first time highlights the contribution of environmental contaminants to the development of cancer.^1^ The panel also points out the great need for increased research on environmental risk factors. In a letter to the President that prefaces the report, the panel wrote that “the true burden of environmentally induced cancer has been grossly underestimated.”

The PCP was established in 1971 by the National Cancer Act, the first salvo in former President Nixon’s “war on cancer.” The panel annually reports to the president on the activities of the National Cancer Program, which Jennifer Burt, special assistant to the PCP, describes as “anything that has to do with cancer in the United States.” Current panelists are Margaret Kripke of the University of Texas MD Anderson Cancer Center and LaSalle D. Leffall of Howard University College of Medicine, both appointed by George W. Bush; an open third position awaits appointment by the Obama administration, Burt says.

Past PCP reports have focused on the contribution of lifestyle to cancer, but Kripke says those reports were criticized for not reviewing the contribution of environmental exposures. The panel therefore chose to dedicate this report to environmental risk factors. In developing the report, the panel reviewed more than 400 scientific reports and heard testimony from 45 invited experts at four public meetings.

The report outlines research on consumer products, combustion by-products, and agricultural chemicals used in residential and commercial landscaping. It highlights cancer attributable to radiation and points out that military activities and unnecessary medical X rays are sources of exposure that can increase cancer risk, especially among children.

Although 60% of U.S. cancer deaths are attributed to lifestyle factors such as smoking, lack of exercise, and poor diet,^2^ the factors contributing to the remaining 40% are a mystery, Kripke says. But the panel did not attempt to characterize the percentage of cancers that might be linked to environmental exposures. “We don’t have any real idea of the contribution of environmental factors to human cancer,” Kripke says. The report points out that most cancer research focuses on genetic and molecular mechanisms behind the disease.^1^

Several environmental scientists were relieved to see the report take such an honest tone about the need for research. “They really point out where we have huge gaps of data,” says Deborah Swackhamer, a professor of environmental chemistry at the University of Minnesota and chair of the U.S. Environmental Protection Agency’s independent Science Advisory Board. “I think the science they used to back up the report is very mainstream,” she adds.

The American Cancer Society (ACS) agrees with 85–90% of the panel’s report, says Otis Brawley, ACS chief medical officer. Yet Brawley and other cancer researchers fear the emphasis on environmental factors may divert the general public from making positive lifestyle changes at a time when an estimated 41% of Americans will develop cancer during their lives and 21% will die of the disease.^3^ Michael J. Thun, vice president emeritus of epidemiology and surveillance research for the ACS, says, “It would be unfortunate if the effect of this report were to trivialize the importance of other modifiable risk factors that, at present, offer the greatest opportunity in preventing cancer.”^4^
